# Outdoor smoking in Nigeria: prevalence, correlates and predictors

**DOI:** 10.1186/s12889-019-7601-8

**Published:** 2019-10-21

**Authors:** Victor Lasebikan, Tiwatayo Lasebikan, Samson Adepoju

**Affiliations:** 1 Department of Psychiatry, College of Medicine, University of Ibadan, PMB 5116, Ibadan, Nigeria; 2 Federal Neuropsychiatric Hospital, Yaba, Lagos, Nigeria; 3 Department of Psychiatry, University College Hospital, Ibadan, Nigeria

**Keywords:** Open-place smoking, Pack-year, Depression, Alcohol, High-income

## Abstract

**Background:**

There is a lack of data on smoking in outdoor-open bars in Nigeria that may translate into effective legislation on public smoking.

**Method:**

This study determined the prevalence, demographic and clinical correlates as well as predictors of smoking among a community sample of 1119 patrons of open place bars in Ibadan, Nigeria. Data on current smoking was obtained using the Alcohol, Smoking and Substance Involvement Screening Test (ASSIST), while smoking intensity was calculated using the Pack-Year. Prevalence of alcohol use was determined using the Alcohol Use Disorders Identification Test (AUDIT), while depression was diagnosed using the Mini International Neuropsychiatry Interview (MINI). Analysis was carried out by SPSS version 20.0 software using Chi square statistics, t test and ANOVA, and was set at 95% confidence interval.

**Results:**

Prevalence of current smoking was 63.8% and the mean pack years of smoking of all respondents was 19.38 ± 17.16 years. Predictors of outdoor smoking were depression OR = 1.41, 95% CI (1.09–1.83) and alcohol use OR = 2.12, 95% CI (1.44–3.13).

Predictors of high pack years were depression OR = 1.47, 95% CI (1.08–2.01), being married, OR = 1.78, 95% CI (1.29–2.45), high income, OR = 1.95, 95% CI (1.42–2.68) and alcohol use OR = 2.82, 95% CI (1.51–5.27). There was no significant relationship between stage of readiness to quit smoking and mean pack years of smoking, F = 0.3, *p* = 0.5.

**Conclusion:**

The high prevalence of outdoor smoking in the sample calls for urgent public health initiatives for intervention. Thus, outdoor bars are potential tobacco use intervention sites to minimize the health consequences of smoking.

## Background

Tobacco use is a leading cause of morbidity and mortality all over the world and in Sub-Saharan Africa, and is currently in stage 1 of the tobacco epidemic continuum [1–3], Stage 1, being the onset of a rising smoking epidemic, but the prevalence still low (< 15%) [1]. In the past several decades, Western European countries reported the highest tobacco consumption rate (37% prevalence among men and 25% among women) [4], however, the trend had changed in the past two decades, with cigarette consumption on the decline while it had increased in Africa.

The rising prevalence of tobacco use in Nigeria might be linked to the uncensored marketing strategies of tobacco companies and poor tobacco control policies in the country [5, 6].

For example, according to the tobacco control act in Nigeria of 2015, outdoor smoking in recreational centre of any form is prohibited [7]. Unfortunately, implementing the regulations has not been applauded by both Houses of the National Assembly [6].

For instance, in Nigeria, cigarette importation has grown more than a hundred folds between 1970 and 2000 [8]. In addition, in Africa, except for Egypt, and South Africa, Nigeria has the largest tobacco market [8].

Thus, Nigeria continues to dominate in smoking epidemic. Estimates show that smoking increases the risk for coronary heart disease by 2 to 4 times, stroke by 2 to 4 times, men developing lung cancer by 25 times and women developing lung cancer by 25.7 times [9]. Smoking is also associated with other chronic diseases such as depression [10] and alcohol abuse [11, 12]. Currently, there are several hypotheses that explain the association between smoking and depression. One is the self-medication hypothesis that postulates that individuals with depression smoke to alleviate their symptoms [13]. The alternative hypothesis is that smoking increases an individual’s susceptibility to environmental stressors because it dysregulates the hypothalamic–pituitary–adrenal system, resulting in hypersecretion of cortisol, thereby leading to depression [14].

Smoking also has documented ethnic [15], sex [16], age [17] and rural-urban [18] variability.

Strikingly is a rapid epidemiological shift of recreational activities in Nigeria to outdoor-open spaces, such as motor-parks, by the road sides, the majority of which are unlicensed premises for such activities [19]. Owners of such open-places entice consumers by providing sources of entertainment, thereby encouraging patrons to smoke without any restriction.

In some countries of the world, outdoor smoke-free policies, despite their criticism have become popular and socially accepted, with public support over time [20] and in all, there are 78 countries in the world with outdoor/quasi-outdoor spaces smoking restrictions [21].

However, in developing countries such as Nigeria, the efforts aimed at reducing outdoor smoking is thwarted by inability to enforce drug policy and most smokers favour [6].

Therefore, our aim in the present study was to determine the prevalence and predictors of outdoor smoking in selected open social joints in Nigeria. We also assessed the relationship between readiness to quit smoking and pack years of smoking. Furthermore, we assessed the association between smoking and depression as well as the association between smoking and alcohol consumption. This is because a key finding from our recent report on outdoor drinking among the same population shows an association between depression and alcohol [22]. In this study, we defined open spaces as roofless joints such as motor-parks, by the roadsides or street corners.

## Methods

### Setting of study and background information on the area

This study is part of a larger study on “alcohol and drug use in open recreational/social joints” in Nigeria. The methodology had been previously described [19], briefly stated, this was a descriptive cross-sectional survey carried out in Ibadan, Nigeria in July 2015. Ibadan is a major city in Nigeria, and has a population of over 2.5 million people according to 2009 census [23].

### Sampling technique and procedure

**Fig. 1:**
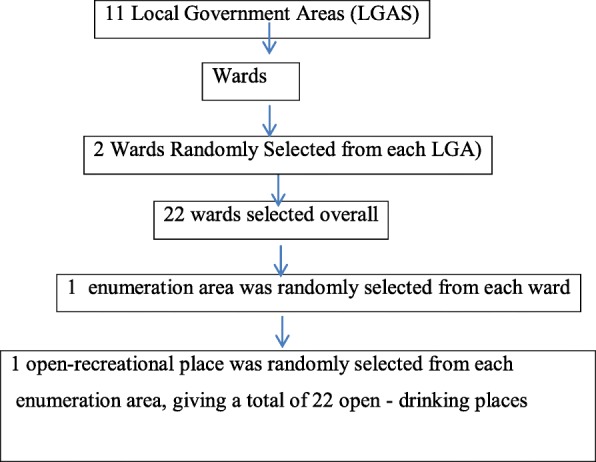
Sample Selection Flow Chart

In this study, we selected 1119 participants through a systematic sampling method from the 11 local governments in Ibadan, after which 2 wards were randomly selected from each local governments, a ward being a local authority unit so designed for electoral purposes [24] and them one enumeration area randomly selected from each of the ward. An open recreational place was then randomly selected thereby yielding a total of 22 open recreational places from the 11 local governments. As stated earlier, this same sampling method was adopted in our previous study [19] and is illustrated in Fig. 1. The list of licensed recreational places was obtained from the state ministry of commerce and were then classified into urban, semi-urban and rural category based on local government classification. Therefore, the amount of fund allocation is directly proportional to the development of a local government and is also a function of the socioeconomic status of the state. Therefore, urban areas are cities with a higher fund allocation [25]. This sampling method increases accessibility grassroots information within the community as was similarly adopted in another study, not part of the current study shisha smoking in selected nightclubs in Nigeria [26].

#### Sample size

A minimum sample of 384 was obtained using the formula for a descriptive cross-sectional study n = Z^2^pq/d^2^, where Z = 1.96, p = anticipated prevalence of outdoor smoking in Nigeria, taken (50%) in the absence of an earlier data on this population, q = 1-p = (50%), d = 0.05 (precision at 95% CI) [27]. However, 10% of the calculated minimum sample of 384 was added yielding 422. We counted 1, 617, but were able to approach 1507 for interview. This was because 110 individuals either intoxicated with alcohol or had a language barrier. In this study, we obtained consent from 1393 patrons, who were all interviewed as soon as the recreational areas opened operation at about 6 pm. In each day of the interview, interview lasted an average of 120 min. Interviewing 1393 participant rather than 422 increased the power of the study.

The data collectors had no personal relationship with the owners of the premises to avoid information bias, although permission for the study was sought from them.

Thus, we interviewed all subjects who gave us their consent using a purposive sampling method until they had all been interviewed.

All the tables in each of the recreational places were numbered, after which the patrons sitting round each table were allocated numbered tallies. An effort was made to ensure that tallied numbers were not duplicated by using continuous numbering until all consenting participants were allocated numbers. We started by interviewing table number 1 and continued until all participants in all the numbered tables were interviewed. The participant with tally number 1 was first interviewed and the research assistants continued consecutively until all patrons sitting round a table were interviewed. The response rate was 92.4%.

### Data collection

Experienced senior registrars in psychiatry who had received prior training in the research protocol and who had been involved in field surveys were used as interviewers. Data collection was supervised by three supervisors and efforts were made to ensure the correct implementation of and full adherence to the research protocol.

### Measures

#### Sociodemographic characteristics

Information about sociodemographic characteristics such as age, sex, marital status, occupation, education, income, and dwelling area (classified into urban, semi-urban and rural) was obtained using a sociodemographic questionnaire.

#### Prevalence of smoking

Information regarding on current smoking were obtained according to the Alcohol, Smoking and Substance Involvement Screening Test (ASSIST) [28]. The ASSIST has been previously validated and used in Nigeria [29].

In the ASSIST questionnaire [29], lifetime prevalence of tobacco use is obtained from Q1: “In your life, which of the following substances have you ever used (non-medical use only)?”

Current prevalence of tobacco use is obtained from Q2: “In the past 3 months how often have you used the substances you mentioned outdoor specifically at this open bar?” Responses were “never,” “once or twice,” “monthly,” “weekly,” and “daily/almost daily.”

For the purpose of this study, any response, other than never, was considered to be current smoking [30].

#### Smoking intensity

Smoking intensity was derived using the Pack-Year [31]. We asked the questions: “On average, how many cigarettes did you smoke per day? There are 20 cigarettes in a pack,” and “For how many years have you smoked?” to compute the Pack-Year. The Pack Year is computed using the formula: (no of years of smoking * Average no of cigarette smoked per day) ÷ 20 cigarettes in a pack.

#### Readiness to quit

We assessed respondent’s readiness to quit: pre - contemplation, contemplation, preparation, action, and maintenance [32]. The profoma used to determine readiness to quit was similar to our previous study on shisha smoking in selected nightclubs in Nigeria [26].

The questions asked were as follows:

Pre-contemplation: Do you have any intention to change cigarette smoking in the foreseeable future? Response was either “yes” or “no”.

Contemplation: Are you aware that your cigarette smoking is a problem and are you seriously thinking about overcoming it, but have not yet made a commitment to take action? Response was either yes” or no”.

Preparation: Are you intending to stop cigarette smoking in the next month? The response was either “yes” or “no”.

Action: Have you successfully achieved abstinence for a period of from one day to six months in the past year? The response was either “yes” or “no”.

Maintenance: Have you prevented a relapse to consolidate the gains attained during cigarette abstinence? The response was either “yes” or “no”.

#### Prevalence of alcohol use

Prevalence of alcohol use was obtained by using the Alcohol Use Disorders Identification Test (AUDIT). The AUDIT is a simple method of screening for excessive drinking and to assist in brief assessment. It has cross cultural reliability across gender and age [33]. The AUDIT is very brief, rapid and flexible. Questions 1 to 3 captures hazardous alcohol use, 4 to 6, dependence and 7 to 10, harmful use [34].

Each question in the AUDIT is usually scored from 0 to 4, 0 indicating “never”, 1 indicating (less than monthly), 2 indicating (monthly), 3 indicating (weekly) and 4 indicating (daily or almost daily). Questions 9 and 10 are rated 0, 2 and 4 (from left to right), because they have only 3 responses. A total score of 0 signifies an abstainer, 1–7 indicates low risk users, 8 and above indicates a likely alcohol use disorder. For the purpose of the present study, a score of 1 and above indicates alcohol use. We merged both low and high risk drinkers together under the assumption that the combined use of tobacco and alcohol is present even in light drinkers because of their reinforcing effects [35].

#### Major depressive episode

The diagnosis of current major depressive episode was made using the Mini International Neuropsychiatry Interview (MINI). The MINI is a brief structured interview that generates Axis I psychiatric diagnoses DSM-IV and ICD10 criteria. The MINI is a short structured diagnostic interview, developed jointly by experts in the United States and Europe, to meet the need for a short but accurate, structured psychiatric interview for multicenter clinical trials and epidemiology studies and to be used as a first step in outcome tracking in non-research clinical settings [36, 37].. The MINI has cross cultural validity and has been used in several studies in Nigeria.

#### Pre-test

All instruments of data collection were pre-tested using 150 participants (not part of the study sample) and found that the instruments had good acceptability in terms of willingness to participate, attitude towards the contents of the instruments, satisfaction with participation in the study, perceived consequences of being part of the research, adherence to research protocols and minimal dropout from the study [38].

#### Analysis

The Chi square statistic was used to analyze the associations between smoking and demographic characteristics, while binary regression analysis was used to determine the predictors of outdoor smoking. Mean pack years of smoking was analyzed using the independent t test and the ANOVA. Post-hoc multiple pairwise comparisons were carried out using Tukey test statistic. Predictors of high pack years were determined using multinomial regression. We adjusted for both age and gender because of their known associations with smoking. All analyses were set at 95% confidence interval, *p* <  0.05 and were carried out by the Statistical Package for the Social Sciences (SPSS) version 20.0 software.

## Results

We interviewed 1393 subjects in all; however, data were complete for 1119. The mean age (SD) of all respondents was 39.10 (12.06) years (Not in any Table). The sociodemographic characteristics of the respondents are presented in Table 1.

**Table 1:** Sociodemography of Smokers (*N* = 1119)

Sociodemographic Characteristics		Smoking					
		Yes		No		X^2^	*P*
**Age**	Total	N	%	N	%		
< 24	147	86	66.7	41	32.3	7.1(df5)	0.2
25–34	310	188	60.6	122	39.4		
35–44	328	198	60.4	130	39.6		
45–54	224	152	67.9	72	32.1		
55–64	98	68	69.4	30	30.6		
> 64	32	22	68.8	10	31.3		
Gender							
Male	826	554	67.1	272	32.9	14.5	< 0.001
Female	293	160	54.6	133	45.4		
Marital Status							
Married	634	417	65.8	217	34.2	2.5	0.1
Unmarried	484	296	61.2	188	38.8		
Religion							
Christianity	1010	641	63.5	369	36.5	0.5	0.5
Islam	109	73	67.0	36	33.3		
Ethnicity							
Igbo	255	167	65.5	88	34.6	13.2 (df4)	0.01^BS^
Yoruba	556	337	60.6	219	39.4		
Middle Belt	257	184	71.6	73	28.4		
Hausa	30	15	50.0	15	50.0		
Others (Minority)	21	11	52.4	10	47.6		
In Employment							
Yes	955	609	63.8	346	36.2	0.01	0.9
No	162	104	64.2	58	35.8		
Years of Education							
0	253	166	65.6	87	34.4	10.4 (df3)	0.01 ^BS^
1–6	502	300	59.8	202	40.2		
7–12	286	202	70.6	84	29.4		
> 12	78	46	59.0	32	41.0		
Residence							
Urban	308	213	69.2	95	30.8	5.3	0.02
Rural/Semi-rural	810	501	61.8	310	19.6		
Income							
Low income earner	627	404	64.4	223	35.6	0.2	0.6
High income earner	492	310	63.0	182	37.0		
Depression							
Yes	465	318	68.4	147	31.6	7.2	0.007
No	654	396	60.6	258	39.4		
Alcohol Use							
Yes	995	654	65.7	341	34.3	14.4	< 0.001
No	124	60	48.4	64	51.6		

BS**:** Bonferonni Significant

Out of the 1119 respondents, the prevalence of current outdoor smoking was (63.8%). The mean pack years of smoking of all respondents were 19.38 ± 17.16 years. There was no significant difference in the age distribution of smokers compared with non-smokers X^2^ = 7.1, *p* = 0.2. A significantly higher proportion of smokers were men, X^2^ = 14.5, *p* <  0.001. There was also a significant ethnic variation in the prevalence of smoking X^2^ = 13.2, *p* = 0.01. Prevalence of smoking also significantly vary based on years of education X^2^ = 10.4, p = 0.01 and residence, X^2^ = 5.3, *p* = 0.02. Smoking was also significantly more prevalent among those with depression, X^2^ = 7.2, *p* = 0.007, and also among those who were current drinkers, X^2^ = 14.4, *p* <  0.001.

Predictors of outdoor smoking were depression OR = 1.41, 95% CI (1.09–1.83) and alcohol use OR = 2.12, 95% CI (1.44–3.13) (Table 2).

**Table 2:** Predictors of Smoking

Variables in the Equation Prediction (65.7%)								
	B	S.E.	Wald	Df	Sig.	Exp(B)	95% C.I.for EXP(B)	
							Lower	Upper
Ethnicity								
Igbo						1		
Yoruba	.278	.289	.929	1	.335	1.321	.750	2.325
Middle Belt	.299	.419	.510	1	.475	1.348	.594	3.063
Hausa	−.332	.521	.407	1	.523	.717	.258	1.991
Others (Minority)	−.385	.516	.555	1	.456	.681	.248	1.872
Education Years								
0						1		
1–6	−.494	.290	2.902	1	.088	.610	.346	1.077
7–12	−.032	.409	.006	1	.938	.969	.435	2.158
> 12	−.272	.373	.531	1	.466	.762	.367	1.583
Residence								
Urban						1		
Rural /Semi-rural	−.136	.165	.679	1	.410	.873	.631	1.206
Depression								
Yes	.345	.132	6.830	1	.009	1.411	1.090	1.828
No						1		
Alcohol Use								
Yes	.753	.198	14.432	1	.000	2.123	1.440	3.130
No						1		

Smoking intensity significantly varies according to age, F = 214.01, *p* <  0.001 (Table 3). Post-hoc analysis shows that the difference was partly due to a lower mean pack years of respondents < 24 years of age compared with respondents 45–54 years of age, 55–64 years of age and > 64 years of age, *p* <  0.001 respectively, partly due a lower mean pack years of respondents 25–34 years old compared to those 35–44 years, 45–54 years, 55–64 years and > 64 years, p <  0.001 respectively, and also partly due to a lower pack years of respondents 35–44 years compared to those 45–54 years old, 55–64 years old and also > 64 years old, p <  0.001 respectively (Not in Table 1).

**Table 3:** Sociodemographic Characteristics by Smoking Intensity (*N* = 714)

Sociodemographic	Pack Years				
Characteristics					
Age	N	Mean	SD	Statistics	P
< 24	86	10.59	10.21	214.01^F^ (df5)	< 0.001
25–34	188	8.47	8.03		
35–44	198	13.85	6.83		
45–54	152	27.43	13.09		
55–64	68	46.72	14.48		
> 64	22	56.68	25.34		
Gender					
Male	554	20.50	17.56	3.2 ^t^	0.01
Female	160	15.52	15.09		
Marital Status					
Married	417	20.63	16.84	2.4 ^t^	0.017
Unmarried	296	17.53	17.41		
Religion					
Christianity	641	19.60	17.26	0.99 ^t^	0.31
Islam	73	17.50	16.25		
Ethnicity					
Igbo	167	20.43	20.72	2.83 ^F^ (df 4)	0.02
Yoruba	337	20.80	16.91		
Middle Belt	184	15.81	13.17		
Hausa	15	17.91	13.54		
Others (Minority)	11	21.79	22.28		
In Employment					
Yes	609	19.13	16.92	0.99 ^t^	0.32
No	104	20.93	18.57		
Years of Education					
0	166	20.23	20.38	3.48 ^F^	0.016
1–6	300	20.82	17.06		
7–12	202	16.12	13.31		
> 12	46	21.24	18.67		
Residence					
Urban	213	17.85	15.77	−1.55	0.1
Rural/Semi-rural	501	20.04	17.69		
Income					
Low income earner	404	23.15	18.75	5.2 ^t^	< 0.001
High income earner	310	16.49	15.23		
Depression					
Yes	318	21.31	17.53	2.7 ^t^	0.007
No	396	17.84	16.72		
Alcohol Use					
Yes	654	18.82	21.88	2.2 ^t^	0.03
No	60	14.72	21.88		

F: ANOVA; t: t test

Mean pack years was also significantly higher in men compared with women, t = 3.2, *p* = .0.01 and in married respondents, t = 2.4, *p* = 0.07. There was also a significant ethnic difference in the mean pack years, F = 2.83, *p* = 0.02 (Table 3). Post-hoc multiple pairwise comparisons indicate that the difference was due to a higher mean pack years of respondents of Yoruba ethnicity compared with those from the middle belt, *p* = 0.013 (Not in Table 3).

There was also a significant difference in the mean pack years of respondents according to their years of education, F = 3.48, *p* = 0.016 (Table 3). Post-hoc multiple pairwise comparisons indicate that the difference was due to a higher mean pack years of respondents with 1–6 years of education compared with respondents with 7–12 years of education, *p* = 0.014 (Not in Table 3).

Mean pack years was also significantly higher among high income earners, t = 5.2, *p* <  0.001, among respondents with depression t = 2.7, *p* = 0.007 and those who were alcohol users, t = 2.2, *p* = 0.03 (Table 3).

Predictors of high pack years were depression OR = 1.47, 95% CI (1.08–2.01), being married, OR = 1.78, 95% CI (1.29–2.45), high income, OR = 1.95, 95% CI (1.42–2.68) and alcohol use OR = 2.82, 95% CI (1.51–5.27) (Table 4).

**Table 4:** Predictors of High Pack Years

Variables	B	Std. Error	Wald	df	Sig	Exp(B)	Lower Bound	Upper Bound
Intercept	.510	.754	.458	1	.499			
Depression								
Present	.387	.160	5.852	1	.016	1.472	1.076	2.013
Absent						1		
Marital Status								
Married	.574	.164	12.186	1	.000	1.775	1.286	2.450
Unmarried						1		
Ethnicity								
Igbo	−.440	.673	.427	1	.513	.644	.172	2.409
Yoruba	−.019	.657	.001	1	.977	.981	.271	3.554
Middle Belt	−.694	.715	.942	1	.332	.499	.123	2.029
Hausa	−.661	.919	.517	1	.472	.516	.085	3.128
Others (Minority)						1		
Years of Education								
0	.149	.430	.120	1	.729	1.161	.500	2.695
1–6	.433	.450	.926	1	.336	1.542	.638	3.728
7–12	.292	.572	.260	1	.610	1.339	.436	4.111
> 12						1		
Income								
High	.670	.161	17.230	1	.000	1.954	1.424	2.681
Low						1		
Alcohol Use								
Yes	1.038	.318	10.624	1	.001	2.823	1.512	5.268
No						1		

The highest proportion of smokers (82.5%) were in the pre-contemplation stage and only 4.6% were in the preparation stage. There was no significant relationship between stage of readiness to quit smoking and mean pack years of smoking, F = 0.3, *p* = 0.5 (Table 5).

**Table 5:** Stage of Readiness to Quit and Mean Pack Years of Smoking (N = 714)

Stage	n	%	Mean	SD	F	*P*
Pre-contemplation	589	82.5	19.62	17.55	0.3	0.5
Contemplation	92	12.9	18.04	16.15		
Preparation	33	4.6	19.01	12.52		
Action	–	–	–	–		

## Discussion

This study evaluated the prevalence and correlates of outdoor smoking in open recreational locations in Nigeria. To the best of our knowledge, this is the first study that accessed outdoor smoking in such setting in Nigeria.

### Prevalence of smoking

We found that 63.8% were current smokers. This figure is much higher than the smoking prevalence reported in Nigeria (20.6%) [30], India (21%) [39], Canada (16%), and America (20%) [40]. However, compared with smoking prevalence in similar social settings such as bar, night clubs and gaming events, our result is close to the 70% reported by Trotter and colleagues in Australia [41]. Studies have generally indicated that bar attendance and nightclubs are a nexus for risky behaviour across all age groups, including smoking and drinking [42, 43].

### Sociodemography and smoking

Contrary to previous reports [30, 44], our univariate analysis shows that age, sex, employment, marital status, and income level were not associated with smoking. It is likely that different individuals with heterogeneous demography congregate at bars and nightclubs to smoke and drink irrespective of their [43].

However, contrary to previous research findings associating low education with smoking [18], we found high education to be associated with smoking. We also found smoking to be associated with urban areas. Most studies have highlighted that smoking is more prevalent in rural areas [45]. As pointed out earlier, these associations were lost after regression analysis.

The potential explanation for these paradoxical demographic associations could be difference in the study population. While the current study was carried out among patrons of outdoor bars, other studies with whom the present study is compared are general population survey/household surveys [30, 40, 44].

Consistent with previous literatures [30, 40], smokers in our sample comprised of predominantly men. This may be because men are more likely to be involved in risk taking behaviours such as drinking and smoking [46], men also strive for leadership and sexual prowess [47].

Our univariate analysis also shows significant ethnic disparities in smoking rate. This is consistent with reports from Nigeria [48] and also from other parts of the world [49]. However, the association was lost after regression analysis.

### Smoking and depression

In line with previous reports [50, 51], we found a significant association between smoking and depression. This observation could be explained by the self-medication hypothesis [13], that smoking causes depression [14], or could also be a product of shared genetic risk factors [52]. Nevertheless, the high prevalence of depression among the smokers in this sample calls for attention because smoking is a risk factor for suicide [53], so also is depression [54].

### Smoking and alcohol use

Consistent with previous reports [11, 12], we also found that smoking was associated with alcohol use. It is conceptualized that the setting of smoking, such as bars and open recreational clubs is potential places where smoking and drinking is promoted by marketers [43].

Concurrent use of alcohol and tobacco is particularly salient, given the increased the risk of various forms of cancer, cardiovascular diseases and is predictive of illicit drug use [55].

### Pack years

Our present investigation shows that smoking intensity heightened with increasing age. Indeed, respondents who were above 54 years of age had over 27 pack year smoking history. To corroborate this, previous reports showed that those who had 30 *pack*-*years* history of smoking were between the *ages* of 55 and 80 [56, 57]. Unfortunately, only 4.5% of the smokers were prepared to quit smoking.

As expected, we observed that mean pack years was lower in women. This may be because women generally smoke fewer cigarettes per day and have lower nicotine dependence [16, 58], or because of social disapproval in this part of the world. However, we noted that the mean pack year was higher among those who were married. A potential explanation is that marriage is a function of age; therefore married respondents are expected to have higher mean pack years of smoking because they are more likely to be older.

Regarding ethnicity, education and pack years of smoking, although there were significant associations during univariate analysis, these associations were lost after regression analysis.

Notable is the significant association between high-income and high pack years. This may be due to the ability of high income earners to have the continued economic strength of purchasing cigarettes over the years. It has been argued that affordability of cigarette is an important factor in promoting smoking. Specifically the Global Tobacco Economics Consortium [59] found that a 50% increase in cigarette prices will lead to significant smoking cessation in 13 middle-income countries.

### Alcohol

The association between high pack years of smoking and alcohol consumption suggests that the co-use of tobacco and alcohol goes beyond experimentation. Indeed a temporal association has implications for the development of tobacco related morbidity and mortality [60], and chronic exposure to both alcohol and tobacco has been found to increase the risk of cancers of the lung [61], mouth, throat, oesophagus, and upper aerodigestive tract [62].

### Depression

In support of a previous report that found a significant association between depression and higher mean pack years of smoking [63], we found that depression was a predictor of pack years of smoking. This suggests that depression and cumulative smoking may be related, although the design of the present study could not explain the direction of the association.

### Pack years and readiness to quit smoking

The finding that the stage of readiness to quit smoking was not associated with mean pack years is of utmost public health attention. So also is our finding that over 90% of these smokers were not yet prepared to quit. Our data deductively serves to guide and stimulate additional research for the development of country specific tobacco control programs across all ages, given the public health importance of tobacco-related diseases such as cancers, cardiovascular diseases and diabetes [64, 65]. Also important is the issue of second hand smoking (SHS), given that non-smokers usually report SHS exposure in most outdoor settings in which smokers report smoking [66].

### Policy and research implications

The present study has implications for prevention of cancers and other diseases associated with smoking. Public health initiatives need to recognize that bars and public drinking places may create unique opportunities for cancer and cardiovascular diseases prevention.

To corroborate this, anti-smoking interventions for bar patrons have been associated with decreases in binge drinking [67]. The high percentage of non-smokers in the current investigation highlights the need to develop voluntary smoke-free rules in outdoor settings.

An interesting finding in the current study is that sociodemographic correlates and predictors of smoking and pack years are similar in certain areas and dissimilar in others. By implication, future studies require to identify the complex mechanism responsible for the development of heavy smoking of time and implement strategies to address this.

### Strengths and limitations

An important strength of the current study is the large sample size, which has increased the power of the results. However, we are mindful of the ethical dilemma of a large sample size and its financial as well as human resource implication. We also recognize the tendency of large sample size magnifying the bias associated with error resulting from the sampling or study design. Nevertheless, we focus on the representativeness of the study sample, considering that participants were recruited over three months and a large sample has the advantage of increasing the power of the study.

Also the location-bound sampling method employed in the present investigation poses a possible selection bias. The possibility of demand bias should be entertained, given that participants were selected from a list of licensed recreational premises obtained the state government. However, this potential information bias was minimized by interviewing the participants as soon as they arrived at the bars when they were less likely to have been drinking or engaged in other bar activities capable of compromising their attention for the study.

Another limitation to the study is the fact that all participants on a table were sampled - this could result in social desirability bias especially if answers are overheard by their peers.

There are usually methodological shortcomings using location-based sampling because of judgment errors by the researcher. However, this is the only feasible method of data collection that fits the study objective. The descriptive nature of the study also makes a cause-effect relationship difficult to deduct from the study.

## Conclusion

The present study has highlighted the prevalence of smoking and its sociodemographic correlates. We have also demonstrated that depression and alcohol use are associated with cigarette smoking. This population requires smoking cessation intervention because they are in the age bracket of the economically viable and productive segment of the society. There is the need for further research among this population.

## Data Availability

The data sets used and analyzed during the current study are available from the corresponding author on reasonable request.

## Abbreviations

AUD: Alcohol use disorder
AUDIT: Alcohol Use Disorder Identification Test
LGA: Local Government Area
PI: Principal Investigator
